# New approaches to enhance environmental cleaning in an acute tertiary hospital

**DOI:** 10.1186/1753-6561-5-S6-P302

**Published:** 2011-06-29

**Authors:** TK Yuen, LM Lin, LE Choon, GY Yan, CC Sun, CY Chuo Ying, KY Hong

**Affiliations:** 1 Singapore General Hospital, Singapore, Singapore

## Introduction / objectives

Environmental contamination with nosocomial pathogens on the near–patient surfaces and equipment has been associated with its indirect transmission. Thorough, regularly scheduled cleaning and disinfection of the environment is essential to break this transmission. Environmental hygiene is a priority for all healthcare faciltiies. Unfortunately, despite regular cleaning following patient discharge with a multi-drug resistant organism, the environment may still harbor these organisms.

## Methods

A multidisciplinary team was formed to look into different ways to improve environment hygiene. The project was carried out in a 36-bedded medical ward from Jun to Dec 2010.The project looked into enhancement of the environmental cleaning by process re-design. The used of two bucket methods of cleaning the environment with micro-fibre cloths that were well soaked with Mikro Quart or phenolic instead of spraying the disinfectant on the cloths. The 3-days education workshops were tailored to the language and cultural needs of the staff. The topics covered “Highly Touch” surfaces, environmental hygiene and audit methods and finding. Effectiveness of the intervention was evaluated using a fluorescent marker, “Glo-germ” powder at random points in the ward. A checklist was used to monitor the trend of progress. The immediate feedback on the audit finding and action plan was developed for compliance failure to the staff and competency was monitored by direct observation.

## Results

The environmental audit showed an improvement from a median of 64% to 95% following the above intervention (*P*< .001). However, no significant improvement in the nosocomial MRSA infection before and after intervention this could be the period is too short.

## Conclusion

The project intervention has spread hospital-wide and enhances using disposable cloths to reduce time in rinsing and prevent contamination.

## Disclosure of interest

None declared.

